# Plasticity of Life-History Traits and Adult Fitness of Fall Webworm in Relation to Climate Change

**DOI:** 10.3390/insects15010024

**Published:** 2024-01-02

**Authors:** Kailu Wang, Mingxuan Xu, Lvquan Zhao

**Affiliations:** Collaborative Innovation Center of Sustainable Forestry in Southern China, College of Forestry, Nanjing Forestry University, Nanjing 210037, China; kailuwang@njfu.edu.cn (K.W.); xmx3210100088@njfu.edu.cn (M.X.)

**Keywords:** *Hyphantria cunea*, phenotypic plasticity, temperature, life-history, adult fitness

## Abstract

**Simple Summary:**

The fall webworm, *Hyphantria cunea* (Drury) (Lepidoptera: Erebidae, Arctiidae), is a major invasive pest of China. Although it has spread from Dandong City (about 40° N) in Liaoning Province to Nanjing City (about 32° N) in Jiangsu Province and other areas, the effects of temperature changes associated with its geographical spread on its life-history traits and adult fecundity are still unknown. Thus, the current study investigated the effects of larval-rearing temperature on the developmental duration and body mass of larvae, body size, body mass, and energy reserves of pupae, as well as the body size and fecundity of adults. We found that higher larval-rearing temperatures shortened the larval developmental period and increased larval and pupal body mass as well as pupal and adult body size. Higher larval-rearing temperatures also decreased the lipid and carbohydrate content of the pupae but increased their soluble protein content and adult egg production. These results suggest that the life-history traits and fecundity of *H. cunea* are plastic. This information is crucial for better understanding the dispersal adaptation strategies of *H. cunea* and for improving the prediction of population dynamics of this species according to environmental temperatures in different years.

**Abstract:**

Temperature is an important environmental factor influencing the life-history traits of ectotherms. This study investigated the effects of larval-rearing temperature (21, 23, 25, and 27 °C) on the life-history traits and adult fitness of the fall webworm, *Hyphantria cunea*, an economically important invasive pest of China. With the increase in temperature during the larval stage, the larval developmental duration was significantly shortened, and the body mass was significantly increased, as was that of the body mass and size of pupae. The carbohydrate and lipid content of pupae significantly decreased with increasing larval-rearing temperature, whereas the protein content significantly increased. Adult body size and egg production increased significantly with increasing larval-rearing temperature, whereas there was no significant difference in egg diameter. These results indicate that *H. cunea* demonstrates life-history traits plasticity. In addition, the increase in fecundity would maintain a stable population size of *H. cunea* under higher temperatures. Such characteristics could enable *H. cunea* to spread to the more southern, warmer areas of China, posing an increased risk to the forestry industry in these regions.

## 1. Introduction

Phenotypic plasticity refers to the different phenotypes encoded by the genotype of an organism in response to different environmental conditions [1]. Such plasticity facilitates the adaptation of organisms to more variable environments through phenotypic changes, enabling their survival under variable environmental conditions. Phenotypic plasticity in insects has two main manifestations: reaction norm, in which a single genotype of an insect is expressed under different environmental conditions to produce a continuum of phenotypes, and non-genetic polyphenism, in which a single genotype is expressed under different environments to produce a discontinuous phenotypic spectrum [2]. Young larvae are subject to external environmental influences from the onset of cellular development, resulting in phenotypic differences and, to some extent, environmental, genetic, physiological, and developmental constraints [3].

The most common manifestation of phenotypic plasticity is the correlation between body size and ambient temperature [4]. Temperature is a crucial environmental factor affecting the life activities of insects and the most significant of all environmental factors in determining insect growth and development. Insects cannot regulate their body temperature independently and are almost entirely dependent on the environment to provide heat [5]. Within a suitable temperature range, the body temperature and metabolic rate of insects vary with fluctuations in ambient temperature. When food is sufficient, heat is the driving force for insect growth and development. Therefore, higher temperatures promote faster growth and development rates, shorter development times, and smaller body sizes at maturity in insects and other poikilothermic animals, showing a negative correlation between body size and ambient temperature [6,7]. This phenomenon, which is widespread in ectotherms, is known as the temperature–body size law [8]. For example, the body size of *Temnothorax nylanderi* decreases with increasing temperature [9]. In contrast to the above rule, some animals have a positive temperature–body size relationship [10], whereby the body size of the adult is bigger at higher temperatures [11,12]. Most exceptions to this rule are found in insects, such as the borer *Chilo suppressalis,* which, when reared at four different temperatures, showed the maximum body mass at the highest temperature [13].

For insects with mouthparts that degrade in the adults, their body size is determined by the number of ecdyses during the larval stage, as well as the growth rate, developmental period, and the amount of food ingested by mature larvae [14]. Therefore, variations in temperature during the larval stage not only bring about changes in growth rate and the developmental calendar, but also changes in adult body size. Adult fecundity is also correlated with body size in that larger body size is correlated with increased fecundity in females, with more mating opportunities in males, and with more access to resources in both sexes [15,16]. In bees, a larger body size results in a larger foraging range [17], while body size is also positively correlated with the amount of pollen a bee is able to carry [18,19]. Therefore, temperature changes during the larval stage not only affect their body size but also the fitness and fecundity of adults.

The fall webworm, *Hyphantria cunea* (Drury) (Lepidoptera: Arctiidae), a major invasive pest in China [20], is native to North America and is widely distributed in the northern United States, southern Canada, and Mexico. *H. cunea* was introduced to Dandong City (about 40° N) in Liaoning Province of China and has now spread to Nanjing City (about 32° N) in Jiangsu Province and other areas. When *H. cunea* was first introduced into China, two generations occurred in one year, whereas, with its spread to the south and in response to climate change, three generations now occur in most areas of China [21]. *Hyphantria cunea* overwinters as a diapause pupa. However, the length of the photoperiod during the larval stage determines whether it enters diapause [22]. This crucial photoperiod is also affected by environmental changes experienced as the species expands its range south in China.

Due to geographic and latitudinal gradients of temperature, *H. cunea* will encounter temperature changes during the spread process. According to the temperature–body size law, *H. cunea* adults are likely to become smaller under higher temperature conditions. Given that insect body size is correlated with adult fitness, the fecundity of *H. cunea* theoretically will decrease as they spread from northern to southern China, negatively impacting their population fitness. However, whether this is the case is currently unknown. Thus, the current study aimed to clarify the impact of larval-rearing temperatures on their life-history traits, especially in body size of the individuals and adult fecundity of *H. cunea*. Such information will be valuable for modeling the predicted spread of the species and for developing suitable control strategies.

## 2. Materials and Methods

### 2.1. Insect Rearing

The fifth and sixth instar larvae of *H. cunea* were obtained from poplar trees (*Populus deltoids*) in Huai’an City, Jiangsu Province, China, in June 2022. The collected larvae were brought back to the laboratory, placed in an insect-rearing box (7 cm × 14.8 cm × 6 cm), and reared on fresh mulberry leaves, replaced daily until pre-pupation. After pupation, the pupae were transferred to another insect-rearing box containing absorbent cotton and moist qualitative filter paper at the bottom to maintain humidity. The pupae were placed on the filter paper until adult emergence. After eclosion, we transferred the adults to the cages (30 cm × 20 cm × 30 cm), and 15 white paper strips were placed in each cage for the adults to lay their eggs. Newly laid eggs were transferred to Petri dishes (7 cm diameter), which were pre-lined with a layer of absorbent cotton; a layer of moist filter paper was placed on top of the absorbent cotton to maintain moisture. The newly hatched larvae were used for the following experiments. The above experiments were carried out under a photoperiod of L:D 16:8 and a temperature of 25 °C.

### 2.2. Experimental Design

*Hyphantria cunea* overwinters as diapausing pupae from October to May of next year. From May to September, the average temperature range is 20 °C to 27 °C in their current districts. In addition, All the larvae died at 31 °C [23].Thus, we transferred the newly hatched larvae to a new box under a photoperiod of 16 L:8 D and temperatures of 21, 23, 25, and 27 °C. The larvae of *H. cunea* were highly aggregated [24] and were therefore reared in groups according to the above method. We replaced the mulberry leaves and checked for larval molting daily. The larvae were separated and individually reared after entering the 5th instar.

### 2.3. Measurement Parameters

The larval developmental duration, larval (5th and 6th instar) body mass, body size and mass of pupae, adult body size, and female fecundity of *H. cunea* were measured in each treatment group. After hatching, the development of larvae was monitored daily to record the time and number of larvae molting until the larvae entered the pre-pupation stage. On the second day after larval development to the fifth and sixth instars, larvae were randomly selected to dry at 65 °C until reaching constant weight before weighing. On the second day after pupation, we measured each pupa’s length and width with a micrometer (0.01 cm) and then weighed the pupa (AL104 Mettler-Toledo; Mettler-Toledo Zurich, Switzerland, d = 0.0001 g) after drying on them a slide glass at 70 °C for 24 h. The dried pupae were used in the following experiments on pupal energy reserves. The sex of each pupa was determined by the presence (female) or absence (male) of a line intersecting the first abdominal sternite [25,26]. After adult emergence, they were treated according to the method described in the insect rearing section. After the females had laid eggs, the right wing, adult body length, and hind leg segment length were measured using the micrometer. The number of eggs was counted from 30 egg clutches randomly selected from each treatment group. The egg diameter was measured using ZEN Imaging Software (version Zen 2, Carl Zeiss, Jena, Germany). The pupal development, adult mating, and oviposition were carried out under a photoperiod of L:D 16:8 and a temperature of 25 °C.

### 2.4. Pupal Energy Reserves

The pupae were placed in 1.5 mL centrifuge tubes, and 20 mg Na_2_SO_4_ and 200 μL of 75% methanol were added. After sufficient homogenization, the grinding rod was rinsed with 600 μL of trichloromethane: methanol (1:1) mixture. After subsequent centrifugation (in all instances, centrifugation was performed at 4 °C, 14,000 rpm for 20 min), the supernatant was transferred to another 1.5 mL centrifuge tube, and the sudprecipitation was added with 300 μL of trichloromethane: formaldehyde (1:1) and then recentrifuged. The supernatant was then transferred to the centrifuge tube with the previous supernatant, which was then mixed, and 500 μL of trichloromethane and 300 μL of 1 mol/L NaCl was added. The mixture was centrifuged, and the supernatant was removed. The lower solution was dried at 70 °C, after which 1000 μL of n-hexane and 500 μL of 1 mol/L NaCl were added. The mix was centrifuged again, and the supernatant was used to determine the total lipids as follows: the lower precipitates were dried at 70 °C, 500 μL of 66% ethanol solution was added, and the mix was centrifuged to dissolve the supernatant fully. The supernatant was then removed, and the lower precipitate was dried at 70 °C. Then, 1000 μL of 10% KOH was added and dissolved for 30 min 100 °C, with shaking every 10 min, after which 50 μL of the solution was removed and mixed with 450 μL of water to determine the protein content. The remaining solution was added to 150 μL of ethanol solution and centrifuged. The solution was allowed to rest for 10 min, the supernatant was removed, and the lower precipitate dried at 70 °C. Finally, the precipitate was dissolved in 400 μL of water at 100 °C and used for the determination of carbohydrate content [27].

The total lipid was determined by the vanillin phosphate method: 50 µL of fat solution was mixed with 1 mL of acetic acid. Next, 1 mL of concentrated sulfuric acid was added and the solution was placed in a boiling water bath for 10 min and then cooled in running water. Next, 2 mL of vanillin phosphate solution was added and left to cool. The absorbance value was determined at a wavelength of 530 nm, and the concentration of lipids was determined by comparing the sample with cholesterol at a known standard concentration.

Carbohydrate content was determined by the anthrone method: 50 µL of the sample was mixed with 950 µL of distilled water, after which 4 mL of anthrone solution was added to the sample in an ice water bath. Next, the solution was placed in a boiling water bath for 10 min, cooled under running water, and allowed to stand at room temperature for 10 min. The absorbance values were determined at a wavelength of 620 nm, and the concentration of carbohydrates was determined by comparing the sample with glucose at a known standard concentration.

The soluble protein content was determined using the Thomas blue staining method: 50 µL of the sample was mixed with 950 µL of distilled water. Next, 5 mL of Khao Maas Brilliant Blue solution was added, shaken, and mixed, and then left to stand for 5 min. The absorbance value was measured at a wavelength of 595 nm. The concentration of soluble proteins in the samples was determined by comparing them with the concentration of standard bovine serum proteins. We used the ratio of the measured lipid, carbohydrate, and protein content to the dry body mass of the whole pupa to express the energy reserves of the pupae. The measured lipid, carbohydrate, and soluble protein contents were used to describe the energy reserves of the pupae in contents per unit of lean body mass (mg mg^−1^).

### 2.5. Statistical Analysis

The experimental data were analyzed in SPSS 22.0 (IBM Inc., New York, NY, USA). Due to the significantly larger body size of females compared to males, female and male were analyzed separately. Larval development duration was analyzed by two-way analysis of variance (ANOVA) with post hoc Tukey’s multiple range test. Larval body mass, pupal body size and mass, and energy reserves were analyzed by ANOVA. ANOVA was also used to analyze the body size, fecundity, and egg diameter of adults.

## 3. Results

### 3.1. Larval Development Duration and Body Mass

Given that larvae were reared in aggregate through to the end of their first–fourth instar, this study separately counted the development duration of larvae at first–fourth, fifth, sixth, and in the entire larval stage. Development duration differed significantly between the temperature treatments (F_3,992_ = 477.1, *p* < 0.001, Table 1), time (instar stage) had an effect on the development duration (F_3,992_ = 8924.1, *p* < 0.001, Table 1), and there was interaction between larvae reared temperature and time (F_9,992_ = 210.2, *p* < 0.001, Table 1). For the first–fourth instars, the larval development duration of the 21 °C treatment group was significantly longer than those of the other three treatment groups (*p* < 0.05 in all cases), and the larval development duration of the 23 and 25 °C treatment groups was similar (*p* > 0.05), but both were significantly longer than that of the 27 °C treatment group (*p* < 0.01). For the fifth instars, the development durations of the 23, 25, and 27 °C treatment groups were similar (*p* > 0.05), but all were significantly shorter than that of the 21 °C treatment group (*p* < 0.05 in all cases). For the sixth instars, the larval development duration of the 21 and 23 °C treatment groups were similar (*p* > 0.05), but significantly longer than those of the 25 and 27 °C treatment groups (*p* < 0.05). The entire larval development duration showed a significant shortening trend with increasing temperature (*p* < 0.01, Table 1).

**Figure 1:**
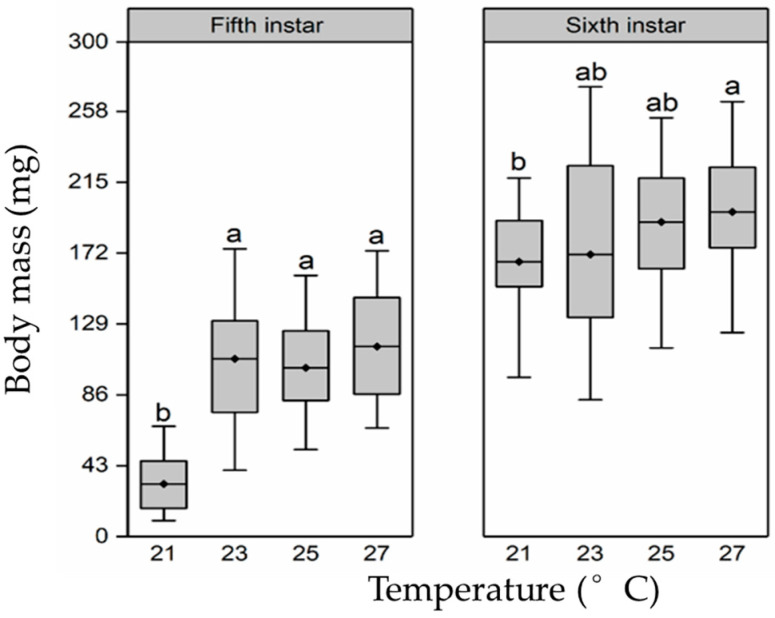
Larval *H. cunea* body mass of fifth and sixth instar larvae at different rearing temperatures. The top and bottom of each boxplot represent the upper and lower quartile, respectively; the horizontal line within the box represents the mean; the whiskers extend to the minimum and maximum values within 1.5× the interquartile range. Different letters indicate statistically significant differences among groups (n = 30).

Based on the body mass analysis, larval-rearing temperature had a significant effect on their body mass (fifth instar, F_3,116_ = 45.1, *p* < 0.01; sixth instar, F_3,116_ = 4.2, *p* = 0.01; Figure 1). The body mass of the fifth instar larvae showed a sharp increase from 21 °C to 23 °C and then maintained a relatively high level in 25 and 27 °C treatment groups. Notably, the body mass of larvae in the 23, 25, and 27 °C treatment groups was significantly higher than that of the 21 °C group (*p* < 0.05 in all cases). Similar to the fifth instar, the body mass of the sixth instar larvae increased at higher temperatures, and the body mass in the 27 °C treatment group was significantly greater than that of the 21 °C treatment group (*p* < 0.05).

### 3.2. Body Mass and Size of Pupae

**Figure 2:**
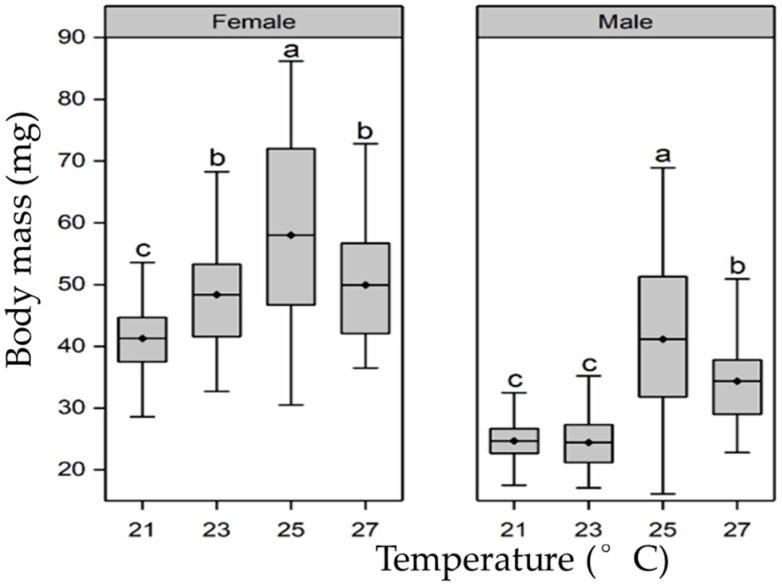
Effect of larval-rearing temperature on the female and male *H. cunea* pupal weight. The top and bottom of each boxplot represent the upper and lower quartile, respectively; the horizontal line within the box represents the mean; the whiskers extend to the minimum and maximum values within 1.5× the interquartile range. Different letters indicate statistically significant differences among groups (n = 30).

Larvae rearing temperature had a significant effect on the body mass of pupae (female pupae, F_3,107_ = 10.7, *p* < 0.01; male pupae, F_3,111_ = 27.7, *p* < 0.01; Figure 2). From 21 °C and 23 °C to 25 °C, the body mass of female pupae showed a sharp increase, with the body mass in the 25 °C treatment group being 1.4-fold and 1.2-fold that of body mass in the 21 °C and 23 °C groups, respectively. However, body mass decreased significantly when the temperature increased from 25 °C to 27 °C, and the body mass in the 27 °C treatment group was only 0.86 times that of the 25 °C treatment group. A similar tendency was found for the body mass of male pupae. 

**Figure 3:**
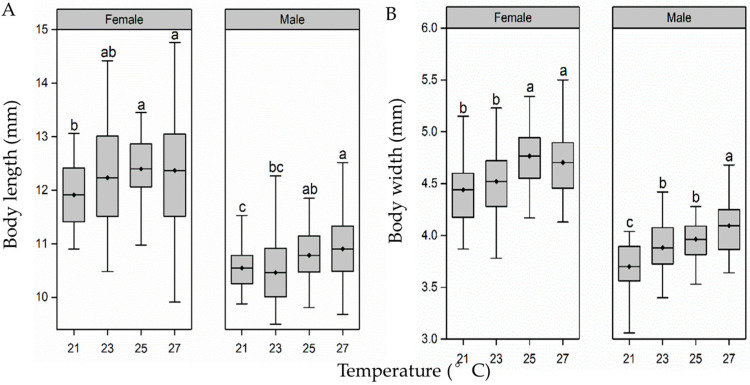
Effect of larval-rearing temperature on the female and male *H. cunea* pupal length (**A**) and pupal width (**B**). The top and bottom of each boxplot represent the upper and lower quartile, respectively; the horizontal line within the box represents the mean; the whiskers extend to the minimum and maximum values within 1.5× the interquartile range. Different letters indicate statistically significant differences among groups (n = 30).

Larvae rearing temperature also had a significant effect on the body size of pupae (length: female pupae, F_3,156_ = 2.7, *p* < 0.05; male pupae, F_3,156_ = 5.0, *p* < 0.01; Figure 3A. width: female pupae, F_3,156_ = 7.7, *p* < 0.01; male pupae, F_3,156_ = 16.6, *p* < 0.01; Figure 3B). The length of female pupae remained stable when the temperature increased from 21 °C to 27 °C, whereas the length of male pupae slightly increased, reaching the maximum in the 27 °C group, being 1.03 times, 1.04 times and 1.01 times longer relative to that in the 21 °C, 23 °C and 25 °C groups. When the temperature increased from 21 °C to 27 °C, the width of female pupae showed a slight increase; the pupal width of the 27 °C group was 1.06, 1.04 and 1.05 times that of 21 °C, 23 °C and 25 °C groups. A similar trend was found for the width of male pupae, which were also widest in the 27 °C treatment group.

### 3.3. Body Composition of Pupae

**Figure 4:**
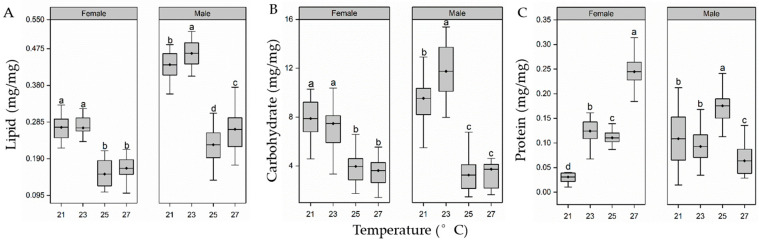
Effect of larval-rearing temperature on lipid (**A**), carbohydrate (**B**), and protein (**C**) content of female and male *H. cunea* pupae. The top and bottom of each boxplot represent the upper and lower quartile, respectively; the horizontal line within the box represents the mean; the whiskers extend to the minimum and maximum values within 1.5× the interquartile range. Different letters indicate statistically significant differences among groups (n = 24–30).

Larvae reared temperature significantly affected the lipid content of pupae (female pupae, F_3,98_ = 127.2, *p* < 0.01; male pupae, F_3,107_ = 216.5, *p* < 0.01; Figure 4A). The lipid content of females remained stable from 21 °C to 23 °C but declined when the temperature increased to 25 °C and 27 °C (*p* < 0.05). Importantly, lipids in the pupae in the 25 and 27 °C groups were significantly less than those in the 21 °C and 23 °C treatment groups (*p* < 0.05). Unlike female pupae, the lipid content of male pupae increased slightly from 21 °C to 23 °C (*p* < 0.05), sharply declined from 23 °C to 25 °C *(p* < 0.05), and then recovered from 25 °C to 27 °C (*p* < 0.05). Notably, there were significant differences in lipid content in pupae between all rearing temperatures (*p* < 0.05 in all cases).

Larvae rearing temperature also had a significant effect on the carbohydrate content of pupae (female pupae, F_3,113_ = 45.7, *p* < 0.01; male pupae, F_3,111_ = 124.6, *p* < 0.01; Figure 4B). Similar to the lipid content of female pupae, the carbohydrate content of females remained stable from 21 °C to 23 °C but declined sharply when the temperature increased to 25 °C and 27 °C. Significantly higher carbohydrate levels were observed in the 21 °C and 23 °C treatment groups compared with the 25 °C and 27 °C treatment groups (*p* < 0.05 in all cases). From 21 °C to 23 °C, the male pupae showed a sharp increase in carbohydrate content, whereas that in the 25 °C and 27 °C treatment groups decreased sharply compared with the 23 °C treatment group, being only 0.31 and 0.37 times, respectively, that of the carbohydrate content in the latter.

In addition, larvae rearing temperature significantly affected the soluble protein content of pupae (female pupae, F_3,113_ = 437.1, *p* < 0.01; male pupae, F_3,108_ = 32.8, *p* < 0.01; Figure 4C). In female pupae, the soluble protein content showed increased sharply from 21 to 23 °C and a slight decline from 23 to 25 °C, sharply increased from 25 to 27 °C again. Comparisons showed 7.9, 1.97, and 2.22-fold higher protein contents in the 27 °C group compared with the groups at 21 °C, 23 °C, and 25 °C (*p* < 0.05 in all cases). Unlike the female pupae, the protein content of male pupae remained stable from 21 to 23 °C, was increased from 23 °C to 25 °C and decreased sharply from 25 °C to 27 °C. The protein content of male pupae in the 25 °C group was 1.62, 1.89, and 2.75 times that in the 21, 23, and 27 °C groups, respectively (*p* < 0.05 in all cases).

### 3.4. Adult Fitness

**Figure 5:**
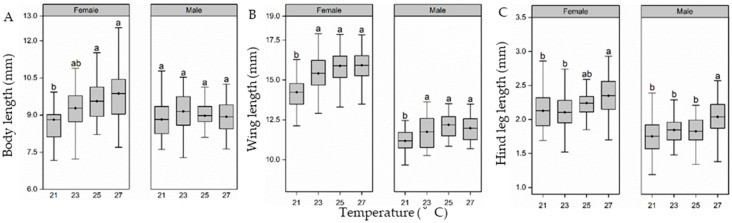
Effect of larval-rearing temperature on the adult *H. cunea* body length (**A**), wing length (**B**), and hind leg length (**C**). The top and bottom of each boxplot represent the upper and lower quartile, respectively; the horizontal line within the box represents the mean; the whiskers extend to the minimum and maximum values within 1.5× the interquartile range. Different letters indicate statistically significant differences among groups (n = 30).

There was a significant difference in the body length of female adults but not of male adults of the groups reared at different larval-reared temperatures (female adult, F_3,116_ = 6.1, *p* = 0.01; male adult, F_3,116_ = 0.9, *p* = 0.43; Figure 5A). The female body length in the 21 °C groups was similar to that in the 23 °C group but was significantly lower than that in the 25 and 27 °C groups. There was a significant difference in the wing length of the adults reared at different larval-reared temperatures (female adult, F_3,116_ = 15.503, *p* < 0.01; male adult, F_3,116_ = 7.113, *p* < 0.01; Figure 5B). The wing length of female adults increased sharply from 21 to 23 °C and was maintained at a relatively high level in 25 and 27 °C, being 1.08, 1.12, and 1.12 times that observed in the 23 °C, 25 °C, and 27 °C treatment groups, respectively relative to the 21 °C group. A similar trend was observed in male adult wing length. There was a significant difference in the length of the hind leg (female adult, F_3,116_ = 5.1, *p* = 0.03; male adult, F_3,116_ = 6.1, *p* < 0.05; Figure 5C). In the analysis of the length of the hind leg segments, those of the females increased gradually from 21 to 27 °C, and the hind leg segments were significantly longer in the 27 °C group relative to the 21 and 23 °C groups (*p* < 0.05 in all cases, Figure 5C). The male adults showed a similar trend (Figure 5C).

**Figure 6:**
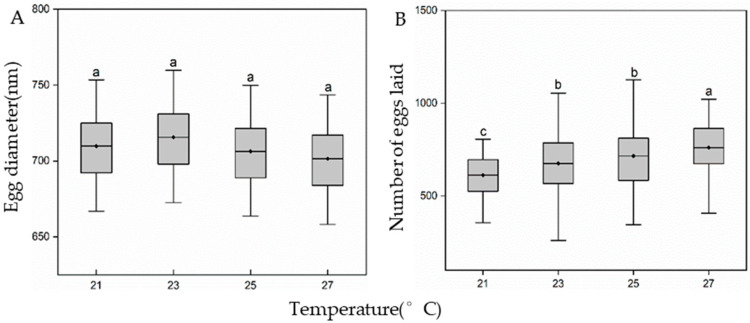
Effect of larval-rearing temperature on the number of *H. cunea* egg diameter (**A**) and egg laid (**B**). The top and bottom of each boxplot represent the upper and lower quartile, respectively; the horizontal line within the box represents the mean; the whiskers extend to the minimum and maximum values within 1.5× the interquartile range. Different letters indicate statistically significant differences among groups (n = 30).

Larval-rearing temperature did not affect egg diameter (F_3,152_ = 2.4, *p* = 0.07; Figure 6A) but had a significant effect on female fecundity (F_3,113_ = 3.3, *p* = 0.02; Figure 6B). Fecundity in the 23 and 25 °C treatment groups was significantly higher than that of the 21 °C treatment group (*p* < 0.05), but significantly lower than that of the 27 °C treatment group (*p* < 0.05).

## 4. Discussion

The current results revealed that elevated larval-reared temperatures shortened the development duration of *H. cunea* larvae and altered their body mass, as well as that of the pupae and the body sizes of pupae and adults. They also increased the soluble protein content of female pupae and female fecundity. However, the carbohydrate and lipid of the pupae did not increase at higher rearing temperatures, and neither did the egg diameter. These results suggest that life-history traits and adult fitness of *H. cunea* are plastic, which can help increase their body size and fecundity in response to climate warming. Similar results have been reported for *Drosophila suzukii*, *Onthophagus taurus*, and *Graphosoma lineatum* (L.) [28,29,30].

The development rate of postembryonic in insects depends on external conditions, such as food availability, diet, and temperature [31], with temperature changes affecting the rate of growth and development, duration of the life cycle, and survival rate [32]. The current results showed that the duration of larval instar became shorter at higher rearing temperatures. Similar results were also reported for *Spodoptera exempta* [33]. For insects with more vulnerable larval stages, such as Lepidoptera, the larval stage might encounter predation by natural enemies such as birds, parasites, or various entomopathogens [34]. Therefore, the shortening of the development duration of *H. cunea* larvae in response to elevated temperatures could reduce their risk of predation under natural environmental conditions and, thus, increase their survival rate.

In the current study, the maximum body mass of pupae and larvae, as well as the maximum pupal body size, occurred at either the highest or intermediate larval-rearing temperatures. This suggests that the body size and temperature of *H. cunea* do not follow the temperature–body size law. Similar results were also found for the Asian corn borer *Ostrinia furnacalis* [12,35]. Most temperature–body size law studies have used only two or three treatment temperatures, which is a limitation in determining the actual thermal response law of the study animals [36]. The current study used four temperatures (21, 23, 25, and 27 °C) and showed that *H. cunea* larval development time decreased at the higher rearing temperatures, with the maximum pupal body size occurring at the highest or intermediate larval-reared temperatures, thus providing a good example of an inverse temperature–body-size law. That the *H. cunea* body mass was maximum at high or intermediate larval-reared temperatures suggests that higher temperatures have a more significant effect on the rate of biomass accumulation than on the rate of development [37]; thus, high temperatures have a more significant effect on the growth rate in this species compared with low temperatures.

The size of an individual insect is a reflection of its energy reserves, with larger individuals usually having more such reserves [38]. Energy reserves in insects are mainly proteins, carbohydrates, and lipids, which provide energy for life activities, such as development and reproduction [39]. The current results showed that larval-rearing temperature not only affected the body size of *H. cunea* but also influenced the pupal energy stores. Lipid and carbohydrate content significantly decreased at higher rearing temperatures. These results might be related to the energy requirements for the basal metabolism of *H. cunea* larvae and pupae and the reproductive development of adults after emergence. When *H. cunea* larvae are exposed to high temperatures, the energy consumed is likely to increase as the metabolic rate increases. Lipids and carbohydrates are the most essential energy sources of insects [40,41]. Therefore, the increase in temperature during the larval stage might accelerate their consumption, leading, in turn, to a decrease in lipid and carbohydrate stores after pupation. A previous study on *H. cunea* diapausing pupae showed that their carbohydrate consumption increased significantly at higher temperatures [42], which further confirms our speculation.

Unlike the lipid and carbohydrate contents that decreased with increasing larval-rearing temperature, the protein contents of female pupae significantly increased, and the protein contents of male pupae significantly increased and then decreased. This phenomenon may be related to the differences in the protein reserve requirements of female and male adults. Given that adult H. cunea has degenerative mouthparts [43], which prevent it from feeding on nutrients after eclosion, the proteins required for adult metamorphosis, reproduction, and flight are derived from those stored during the larval stage. Increased protein stores contribute to increased insect stress resistance, and more energy is used for protein synthesis at high temperatures to combat the increase in entropy at higher temperatures [44]. Unlike males, striking is the requirement for egg production by females, which requires substantial amounts of protein [45]. Therefore, the increased protein content in the female pupae at high temperatures helps to enhance their ability to counteract any stress resulting from those high temperatures. The increase in protein content will also contribute to the female adult fecundity, thereby improving the fitness of the population. The results of adult reproductive capacity increasing with temperature further confirm the above speculation.

Body size is positively correlated with fecundity in most female insects [46]. In addition, the increased length of leg segments on the hind leg of insects helps females to better support a larger abdomen to increase their brood amount [47]. The results of the current study were consistent with the above result. At the higher rearing temperatures, adult body length and hind leg segment length were increased, as was female egg production. These results suggest that increasing temperature contributes to increasing female body size and, thus, female fecundity in *H. cunea*.

The resilience of insect eggs is related to their diameter [48]. The results of this study showed that, although a higher larval-rearing temperature increased female fecundity, there was no significant effect on egg diameter, suggesting that the increase in female fecundity did not come at the expense of either egg diameter or egg resilience. The dispersal ability [49] and flight frequency of insects [50] fluctuate with temperature, and increased temperature will lower the flight ability of insects [51]. In addition, larger wing size individuals flew faster and tended to fly longer distances compared to smaller wing size individuals [52]. The current results showed that adult *H. cunea* had longer wings under high-temperature rearing conditions. Therefore, we speculated that this change in wing length might compensate for their loss of flight ability brought about by high-temperature conditions.

## 5. Conclusions

High temperatures shortened the larval developmental period, increased the body mass of larvae and pupae and body size of adults, and increased soluble protein content in pupae and adult egg production. However, high temperatures decreased the pupal lipid and carbohydrate content. These results suggest that the life-history traits of *H. cunea* show plasticity. Invasive species demonstrate significantly higher phenotypic plasticity than co-occurring non-invasive species [53,54]. Moreover, greater plasticity can enhance the adaptability of invasive species to fluctuating and warming environmental conditions [53,54]. Therefore, it is reasonable to hypothesize that the phenotypic plasticity of *H. cunea* would increase its ability to adapt to novel environments and, thus, increase its dispersal range when it encounters climatic warming as it expands its range further into southern China. However, we only explored the effects of 21, 23, 25 and 27 °C on the life-history traits and adult fitness of *H. cunea* in this study. When *H. cunea* expands its range further into southern China, its larval development stage will face higher temperatures. The effects of temperature exceeding the current experimental design on their life-history traits and adult fitness of *H. cunea* remain to be studied.

## Figures and Tables

**Table 1:** Mean (±SE) development duration of *Hyphantria cunea* larvae at different rearing temperatures.

Rearing Temperature	Development Duration (Days)			
	First–Fourth Instar	Fifth Instar	Sixth Instar	Total Larval Stage
21 °C	18.54 ± 0.29 a	7.08 ± 0.23 a	12.42 ± 0. 3 a	38.04 ± 0.46 a
23 °C	15.51 ± 0.17 b	4.43 ± 0.11 b	9.70 ± 0.26 a	29.64 ± 0.28 b
25 °C	16.13 ± 0.14 b	4.66 ± 0.08 b	7.48 ± 0.17 b	28.27 ± 00.24 c
27 °C	12.95 ± 0.18 c	4.02 ± 0.12 b	7.98 ± 0.25 b	24.95 ± 0.31 d

Different letters in the same instar indicate significant differences in development duration between the temperature treatments (*p* < 0.05, n = 48–86).

## Data Availability

The data presented in this study are available on request from the corresponding author.
